# Consent for withholding life-sustaining treatment in cancer patients: a retrospective comparative analysis before and after the enforcement of the Life Extension Medical Decision law

**DOI:** 10.1186/s12910-021-00644-0

**Published:** 2021-06-17

**Authors:** Yu Jin Chung, Incheol Park, Junho Cho, Jin Ho Beom, Ji Eun Lee

**Affiliations:** Department of Emergency Medicine, Yonsei University College of Medicine, 50 Yonsei-ro, Seodaemun-gu 120-752, Seoul, Republic of Korea

**Keywords:** Life Extension Medical Decision law, Length of hospital stay, Cardiopulmonary resuscitation, Intubation, Continuous renal replacement therapy, Intensive care unit

## Abstract

**Background:**

The Life Extension Medical Decision law enacted on February 4, 2018 in South Korea was the first to consider the suspension of futile life-sustaining treatment, and its enactment caused a big controversy in Korean society. However, no study has evaluated whether the actual implementation of life-sustaining treatment has decreased after the enforcement of this law. This study aimed to compare the provision of patient consent before and after the enforcement of this law among cancer patients who visited a tertiary university hospital's emergency room to understand the effects of this law on the clinical care of cancer patients.

**Methods:**

This retrospective single cohort study included advanced cancer patients aged over 19 years who visited the emergency room of a tertiary university hospital. The two study periods were as follows: from February 2017 to January 2018 (before) and from May 2018 to April 2019 (after). The primary outcome was the length of hospital stay. The consent rates to perform cardiopulmonary resuscitation (CPR), intubation, continuous renal replacement therapy (CRRT), and intensive care unit (ICU) admission were the secondary outcomes.

**Results:**

The length of hospital stay decreased after the law was enforced from 4 to 2 days (*p* = 0.001). The rates of direct transfers to secondary hospitals and nursing hospitals increased from 8.2 to 21.2% (*p* = 0.001) and from 1.0 to 9.7%, respectively (*p* < 0.001). The consent rate for admission to the ICU decreased from 6.7 to 2.3% (*p* = 0.032). For CPR and CRRT, the consent rates decreased from 1.0 to 0.0% and from 13.9 to 8.8%, respectively, but the differences were not significant (*p* = 0.226 and *p* = 0.109, respectively).

**Conclusion:**

After the enforcement of the Life Extension Medical Decision law, the length of stay in the tertiary university hospital decreased in patients who established their life-sustaining treatment plans in the emergency room. Moreover, the rate of consent for ICU admission decreased.

## Background

Cancer is one of the three leading causes of death in South Korea, with more than 210,000 newly diagnosed cases every year [1, 2]. In 2020, nearly 10 million people died from cancer worldwide, with approximately one in six deaths being due to cancer [3]. The death burden due to cancer is heavy despite early diagnosis and prompt treatment [4]. Cancer patients receive rigorous chemotherapy and thus, incur high treatments costs and experience prolonged hospital stays compared to patients with other illnesses [5]. Therefore, it is important for cancer patients to make decisions regarding their direction of treatment, considering an improved quality of life and how they wish to spend the rest of their lives. That is, patients have the right to refuse futile life-sustaining treatments and such decisions should be made after thorough discussions between the patient, their family, and the involved medical professionals. In addition, the suspension of life-sustaining treatments must be guaranteed by an unbiased social system in consideration of the relevant ethical aspects.

However, in practice, patients in Korea are rarely involved in the decision to refuse life-sustaining treatments. Several studies on terminal cancer patients have shown that family members and medical professionals make this decision without sufficient discussion with the patient [6]. Furthermore, until now, there have been no laws pertaining to life-sustaining treatments, making it difficult to respect the patients’ right to self-determination.

Consequently, the Life Extension Medical Decision (LEMD) law, regarding the decisions on hospice care, palliative care, and life-sustaining medical care for patients close to death, was enforced on February 4, 2018, as an institutional measure to respect the dignity and rights of patients in South Korea [7]. It provides legal provisions describing the criteria, conditions, and methods for discontinuing futile life-sustaining care. The enforcement of this law resulted in a big controversy in the Korean society because this law respects the patient’s right to self-determination and increases the likelihood of patients dying comfortably while maintaining human dignity.

Since the value of a patient’s life is influenced by social consensus, which differs by culture, regulations related to life-sustaining medical care differ across countries. A similar law was passed in the United States. In 1976, the Natural Death Act was passed in California, United States, and it was the first law to legally consider a patient’s letter of intent to discontinue medical care [8]. In addition, the Patient Self-Determination Act was passed in 1990, respecting patients’ decisions and allowing them the independence to refuse treatment. A recent systematic review reported that between 2011 and 2016, 36.7% of US adults completed advance directives, of whom 29.3% had living wills [9]. Therefore, with the enactment of the LEMD law, changes such as a decrease in the use of life-sustaining treatments and an increase in written consent for orders for Life-Sustaining-Treatment (orders for LST) are expected in South Korea. It is essential to comparatively analyze the types of changes observed in response to the enactment of this law in clinical practice. In particular, previous studies have shown that terminal cancer patients account for the highest proportion of patients receiving life-sustaining treatments (33.2%); therefore, analyzing the influence of the enactment of the LEMD law on the use of life-sustaining treatments in advanced cancer patients would have significant implications in the clinical setting [10].

However, in Korea, research has not been conducted on whether the enactment of the LEMD law has shown practical effectiveness by comparing the conditions before and after the enforcement of this law. Therefore, in this study, we aimed to compare relevant parameters, such as the length of hospital stay; rates of consent to perform cardiopulmonary resuscitation (CPR), intubation, continuous renal replacement therapy (CRRT), and intensive care unit (ICU) admission; and the rate of survival to discharge, before and after the enforcement of the LEMD law in advanced cancer patients who visited the emergency room (ER) of a tertiary university hospital.

## Methods

### Study design

This retrospective single cohort study was conducted at a tertiary university hospital. To compare the effects of the LEMD law, the 1-year periods before and after enforcement were selected as the target research periods; the period of 3 months immediately after the enforcement of the LEMD law was considered the adaptation period and thus, excluded from the study. Therefore, the two final study periods were as follows: from February 2017 to January 2018 (before) and from May 2018 to April 2019 (after).

Primary comparisons were made between these two study periods. The patients from each period were divided into two groups: one group included patients who had signed orders for LST or prepared advance directives before visiting the ER, and the other group included patients who signed their orders for LST or established their life-sustaining treatment plan during their ER visit.

The study data were collected using the Severance Clinical Research Analysis Portal, the clinical data search system used by the hospital. This study was conducted in compliance with the Declaration of Helsinki, and the need for informed consent was waived due to the study’s retrospective design. This study was approved by the Institutional Review Board of the Yonsei University Health System (approval number 4-2020-0630). The need for informed consent was waived by the Institutional Review Board of the Yonsei University Health System (approval number 4-2020-0630).

### Participants

Advanced cancer patients aged over 19 years with stage IV or metastatic cancer who visited the ER of Severance Hospital during the study periods were included in this study.

Overall, the analyzed patients included the following: (1) patients who visited the ER between February 2017 and January 2018, before the enforcement of LEMD law, with signed orders for LST obtained prior to or during the ER visit and (2) patients who visited the ER between May 2018 and April 2019, after the enforcement of the law, with advance directives prepared beforehand or life-sustaining treatment plan established during the ER visit. Among these, cases in which the consent form was signed but the exact information could not be confirmed owing to incomplete item descriptions were excluded.

### Outcomes

We investigated the length of hospital stay as the primary outcome. In addition, the consent rates to perform CPR, intubation, CRRT, and ICU admission were considered as secondary outcomes, to confirm whether the consent rates for life-sustaining treatments and life-sustaining procedures actually reduced with the enactment of the LEMD law. Furthermore, variables such as the time taken from reception to the completion of orders for LST or a life-sustaining treatment plan, length of stay in the ER, transfer from the ER to secondary or nursing hospitals, survival rate in the ER, and medical expenses were also analyzed. “Medical expenses” referred to the total expenses incurred during hospital stay, including ER expenses, during the study periods. The calculations were made collectively by the hospital’s accounting team.

### Description of each consent form

#### Provision of orders for LST before the enforcement of the LEMD law

Orders for LST enforce the abandoning of resuscitation attempts in patients with no prospect of resuscitation, especially in patients with predicted cardiac arrest [11]. This document has an arbitrary format used widely by medical institutions in clinical practice; however, the subject and format are not universal. In addition, before the enforcement of the law, there was no difference in either the title or detailed items between the form received in advance and the one received in the ER.

#### Provision of advance directives after the enforcement of the LEMD law

This form is to be completed at an outpatient department or other medical agencies prior to an ER visit. Any adult aged ≥ 19 years can document his or her intentions for life-sustaining treatments and hospice care directly in preparation for a situation in which the person becomes a patient at the end of life in the future. The document prepared for this purpose is called the advance directive on life-sustaining treatments and should be created after receiving sufficient explanation through the Registry Agencies for Advance Directives designated by the Ministry of Health and Welfare. The legal validity of the created and registered advance directives can be recognized only when they are stored in the database of the life-sustaining treatments information processing system [7].

Provision of a life-sustaining treatment plan after the enforcement of the LEMD law

This form is to be filled out when a patient who has not previously completed advance directives visits the ER. If the patient is conscious, the patient’s preference is noted. If the patient is unconscious, the form is filled out after discussion between the guardian and doctor.

**Fig. 1:**
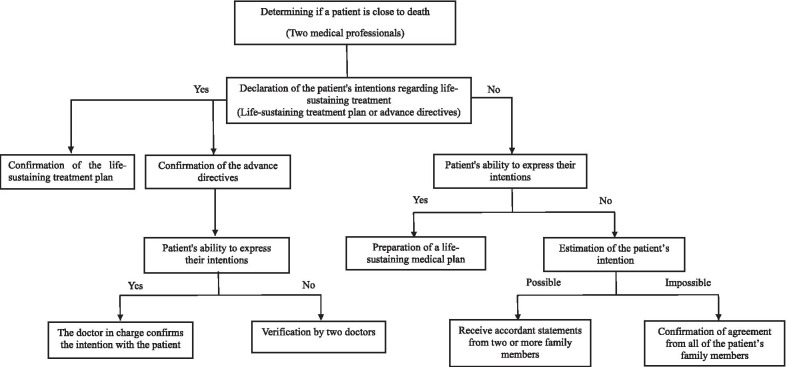
Procedure of decision-making regarding withholding life-sustaining treatment

According to the decision of the terminally ill patient, the doctor-in-charge plans and documents the decisions related to the patient’s life-sustaining medical treatment and hospice care [7], and this process is shown in Fig. 1 [12].

As shown in Fig. 1, if a patient who has not indicated an intention to withhold life-sustaining treatments in advance is unconscious, a decision can be made only after all family members come to a unanimous agreement (Table 1).

**Table 1:** Comparison of details of the consent forms

Orders for LST^a^ (before the enforcement of the LEMD law)	Advance directives or life-sustaining treatment plan after the enforcement of the LEMD law
CPR	CPR
Endotracheal intubation and ventilator care	Ventilator care
CRRT	Hemodialysis
	Administration of anti-cancer drugs
Vasopressor and inotropic agent use	
Increased vasopressor and inotropic agent use	
Transfusion	
Blood test	
ICU care	ICU care

*LST* Life-Sustaining-Treatment, *CPR* cardiopulmonary resuscitation, *CRRT* continuous renal replacement therapy, *ICU* intensive care unit, *LEMD* law Life Extension Medical Decision law

Orders for LST^a^: Before the enforcement of the LEMD law, each hospital had its own independent consent form. The items described above are based on the format used at our hospital

### Statistical analysis

The collected data were analyzed using the SAS software (version 9.4, SAS Inc., Cary, NC, USA). All continuous variables except age did not satisfy the normality assumption; thus, the variables were analyzed using the Wilcoxon rank-sum test, and the results were presented as medians (Q1, Q3). Age was presented as the mean ± standard deviation. Categorical variables were analyzed using the chi-square test (Fisher’s exact test) and presented as counts (%). The Monte Carlo estimation method was used to accurately determine the location of the primary cancer. A *p* value < 0.05 was considered significant.

## Results

**Fig. 2:**
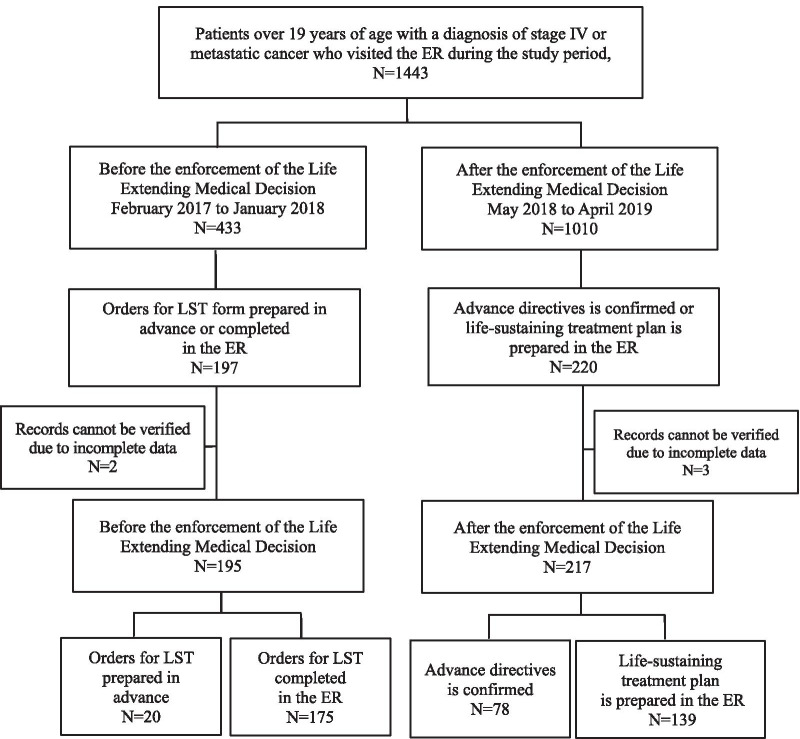
Flow diagram of patient eligibility. *LST* life-sustaining-treatment, *ER* emergency room, *OPD* outpatient department

During the study period, a total of 1443 patients with stage IV advanced cancer or metastatic cancer were admitted to the hospital’s ER. Overall, 433 and 1010 patients visited the hospital before (February 2017–January 2018) and after (May 2018–April 2019) the enforcement of the LEMD law, respectively. Prior to enforcement, 197 patients had prepared orders for LST in advance or in the ER, while after the enforcement of the LEMD law, 220 patients had prepared advance directives before visiting the ER or established a life-sustaining treatment plan in the ER. Of these, five patients (two, before enforcement; three, after enforcement) were excluded from the study due to incomplete information on the forms. Finally, 195 patients presenting before enforcement and 217 patients presenting after enforcement were included in this study. Of the 195 patients presenting before enforcement, 20 had completed orders for LST before visiting the ER and 175 did so in the ER. Of the 217 patients presenting after enforcement, 78 had prepared advance directives before visiting the ER and 139 wrote their life-sustaining treatment plan in the ER (Fig. 2).

The basic characteristics of the patients enrolled in this study before and after the enforcement of the LEMD law are shown in Table 2.

**Table 2:** Patient characteristics before and after the enforcement of the LEMD law

Variables	Group		*p *value
	Orders for LST before enforcement of the LEMD law (n = 195)	Advance directives or life-sustaining treatment plan after enforcement of the LEMD law (n = 217)	
Age (years)	68.3 ± 11.5	63.0 ± 7.7	0.159
Sex, male	107 (54.9)	100 (46.1)	0.178
Primary cancer			0.599
Pancreatic cancer	15 (7.69)	18 (8.3)	
Gastric cancer	24 (12.3)	16 (7.4)	
Head, neck, and oral cancers	6 (3.08)	8 (3.7)	
Liver and intrahepatic bile duct cancers	25 (12.8)	37 (17.1)	
Lung, tracheal, and bronchial cancers	47 (24.1)	46 (21.2)	
Hematologic cancer	1 (0.5)	1 (0.5)	
Esophageal cancer	2 (1.0)	3 (1.4)	
Colorectal cancer	21 (10.8)	36 (16.6)	
Breast cancer	19 (9.7)	14 (6.5)	
Prostatic cancer	3 (1.5)	5 (2.3)	
Uterus, ovarian, and cervical cancers	8 (4.1)	12 (5.5)	
Kidney cancer	5 (2.6)	3 (1.4)	
Bladder cancer	3 (1.5)	3 (1.4)	
Skin cancer	3 (1.5)	6 (2.8)	
Others	13 (6.7)	9 (4.2)	

Data are presented as the mean ± standard deviation or n (%)

*LST* Life-Sustaining-Treatment, *LEMD law* Life Extension Medical Decision law

We compared patients who completed their orders for LST, before or during the ER visit, in the pre-enforcement period with patients who completed their advance directives before the ER visit or created their life-sustaining treatment plan during the ER visit in the post-enforcement period.

After the enforcement of the LEMD law, the time taken to obtain consent forms (orders for LST before enforcement/advance directives or life-sustaining treatment plan after enforcement) was reduced to 3 h and 52 min from 4 h and 33 min (*p* = 0.031); conversely, the length of stay in the ER increased from 13 h and 37 min to 19 h and 55 min (*p* = 0.006). The length of stay in the hospital decreased from 4 to 2 days (*p* = 0.001), along with a decrease in medical expenses (*p* = 0.008). Furthermore, the rate of direct transfers from the ER to secondary hospitals increased from 8.2 to 21.2% (*p* = 0.001), and that of transfers to nursing hospitals increased from 1.0 to 9.7% (*p* < 0.001) (Table 3).

**Table 3:** Comparison of parameters before and after the enforcement of the LEMD law

Variables	Group		*p *value
	Orders for LST before enforcement of the LEMD law (n = 195)	Advance directives or life-sustaining treatment plan after enforcement of the LEMD law (n = 217)	
Time taken from reception to obtaining written consent (min)	273 (117, 501)	232 (0, 683)	0.031
Length of stay in the ER (min)	817 (447, 1705)	1195 (594, 2151)	0.006
Length of hospital stay (days)	4 (1, 10)	2 (0, 8)	0.001
Medical expenses (won)	3,718,989 (1,776,908, 6,629,026)	2,691,195 (1,356,584, 5,347,890)	0.008
Type of ER visit			0.860
Direct visit to the ER without transfer from another hospital	144 (73.9)	155 (71.4)	
Transfer from another hospital	46 (23.6)	56 (25.8)	
Transfer from the OPD	5 (2.6)	6 (2.8)	
Transfer from the ER to a secondary hospital	16 (8.2)	46 (21.2)	0.001
Transfer from the ER to a nursing hospital	2 (1.0)	21 (9.7)	< 0.001
Survival in the ER	173 (88.7)	186 (85.7)	0.363
Life-sustaining treatments and life-sustaining procedures			
Consent to perform CPR	2 (1.0)	0 (0.00)	0.226
Consent to perform intubation	10 (5.1)	11 (5.1)	0.995
Consent to perform CRRT	27 (13.9)	19 (8.8)	0.109
Consent for ICU admission	13 (6.7)	5 (2.3)	0.032
Survival at discharge	56 (28.7)	96 (44.2)	0.001

Data are presented as the median (Q1, Q3) for continuous variables and number (%) for categorical variables

*LST* Life-Sustaining-Treatment, *CPR* cardiopulmonary resuscitation, *CRRT* continuous renal replacement therapy, *ICU* intensive care unit, *ER* emergency room, *OPD* outpatient department

In addition, the consent rate for ICU admission decreased from 6.7% before enforcement to 2.3% after enforcement (*p* = 0.032). After the enforcement of the LEMD law, a large number of patients were found to survive until discharge, and this difference was highly significant (*p* = 0.001) (Table 3).

We performed subgroup analyses according to the place where the consent form was signed. On comparing only those patients who established orders for LST (pre-enforcement) or life-sustaining treatment plan (post-enforcement) in the ER, we found that the time taken from reception to signing the orders for LST was 4 h and 56 min before enforcement, while the same task (creating a life-sustaining treatment plan) took significantly longer (7 h and 46 min) after enforcement (*p* < 0.001). Consistent with the results of the overall comparison, the length of stay in the ER increased from 14 h and 24 min (before enforcement) to 23 h and 11 min (after enforcement) (*p* = 0.003), but the length of hospital stay decreased from 4 to 2 days (*p* = 0.001). The rates of direct transfers from the ER to secondary hospitals and nursing hospitals also increased in the period after enforcement, from 9.1 to 20.1% (*p* = 0.005) and from 1.1 to 10.1% (*p* = 0.001), respectively. The survival rate in the ER decreased from 88.6 to 80.6% after the law was implemented (*p* = 0.049) (Table 4). The proportion of life-sustaining treatments or life-sustaining procedures specified in the orders for LST or life-sustaining treatment plan decreased overall after enforcement, but this difference was not statistically significant. However, there was a significant reduction in the consent rate for ICU admission, from 6.9 to 1.5% (*p* = 0.026) (Table 4).

**Table 4:** Orders for LST before enforcement and life-sustaining treatment plan after enforcement in the ER

Variable	Group		*p *value
	Orders for LST prepared in the ER before enforcement of the LEMD law (n = 175)	Life-sustaining treatment plan prepared in the ER after enforcement of the LEMD law (n = 139)	
Time taken from reception to obtaining written consent (min)	296 (155, 532)	466 (245, 963)	< 0.001
Length of stay in the ER (min)	864 (479, 1750)	1,391 (642, 2,467)	0.003
Length of hospital stay (days)	4 (1, 9)	2 (0, 6)	0.001
Medical expenses (won)	3,782,083 (1,732,649, 6,638,183)	2,755,304 (1,615,569, 4,823,854)	0.076
Type of ER visit			0.208
Direct visit to the ER without transfer from another hospital	134 (76.6)	94 (67.6)	
Transfer from another hospital	36 (20.6)	40 (28.8)	
Transfer from the OPD	5 (2.9)	5 (3.6)	
Transfer from the ER to a secondary hospital	16 (9.1)	28 (20.1)	0.005
Transfer from the ER to a nursing hospital	2 (1.1)	14 (10.1)	0.001
Survival in the ER	155 (88.6)	112 (80.6)	0.049
Life-sustaining treatments and life-sustaining procedures			
Consent to perform CPR	2 (1.1)	0 (0.0)	0.506
Consent to perform intubation	9 (5.1)	6 (4.4)	0.755
Consent to perform CRRT	2 (1.1)	0 (0.0)	0.506
Consent for ICU admission	12 (6.9)	2 (1.5)	0.026
Survival at discharge	53 (30.3)	47 (33.8)	0.505

Data are presented as the median (Q1, Q3) for continuous variables and number (%) for categorical variables

*LST* Life-Sustaining-treatment, *CPR* cardiopulmonary resuscitation, *CRRT* continuous renal replacement therapy, *ICU* intensive care unit, *ER* emergency room, *OPD* outpatient department

Next, we analyzed the results of the following two groups: those who had prepared orders for LST before visiting the ER in the pre-enforcement period and those who had prepared advance directives before visiting the ER in the post-enforcement period. The length of stay in the ER increased from 8 h and 30.5 min (before enforcement) to 16 h and 10.5 min (after enforcement) (*p* = 0.042). The rate of transfer from the ER to a secondary hospital increased from 0.0 to 23.1% (*p* = 0.020). Lastly, the rate of survival to discharge showed a highly significant increase from 15 to 62.8% after the enforcement of the LEMD law (*p* = 0.001) (Table 5). Because the patients included in this comparative analysis had prepared their consent forms (orders for LST or advance directives) in advance, the time taken to complete the consent forms was not evaluated.

**Table 5:** Preparation of orders for LST (pre-enforcement) and advance directives (post-enforcement) before visiting the ER

Variable	Group		*p* value
	Orders for LST before enforcement of the LEMD law (n = 20)	Advance directives after enforcement of the LEMD law (n = 78)	
Length of stay in the ER (min)	510.5 (349.5, 1085)	970.5 (462, 1615)	0.042
Length of hospital stay (days)	5.5 (3, 10)	2 (0, 10)	0.061
Medical expenses (won)	3,593,733.5 (2,439,355.5, 4,899,793.5)	2,167,290.5 (961,601, 6,025,705)	0.133
Type of ER visit			0.025
Direct visit to the ER without transfer from another hospital	10 (50.0)	61 (78.2)	
Transfer from another hospital	10 (50.0)	16 (20.5)	
Transfer from the OPD	0 (0.0)	1 (1.3)	
Transfer from the ER to a secondary hospital	0 (0.0)	18 (23.1)	0.020
Transfer from the ER to a nursing hospital	0 (0.0)	7 (9.1)	0.339
Survival in the ER	18 (90.0)	74 (94.9)	0.599
Life-sustaining treatments and life-sustaining procedures			
Consent to perform CPR	0 (0.0)	0 (0.0)	
Consent to perform intubation	1 (5.0)	5 (6.4)	> 0.999
Consent to perform CRRT	0 (0.0)	4 (5.1)	0.579
Consent for ICU admission	1 (5.0)	3 (3.9)	> 0.999
Survival at discharge	3 (15.0)	49 (62.8)	0.001

Data are presented as the median (Q1, Q3) for continuous variables and number (%) for categorical variables

*LST* Life-Sustaining-treatment, *CPR* cardiopulmonary resuscitation, *CRRT* continuous renal replacement therapy, *ICU* intensive care unit, *ER* emergency room, *OPD* outpatient department

## Discussion

On February 4, 2018, the “Act on Hospice and Palliative Care and Decisions on Life-sustaining Treatment for Patients on End of Life (Life Extension Medical Decision Law)” came into force in South Korea [7]. This was the first law to consider the suspension of futile life-sustaining medical care, which raised a big issue both in the medical field and the social community.

If consent was received in the ER, the time taken to obtain consent for a life-sustaining treatment plan was longer after enforcement of the LEMD law than the time taken to establish orders for LST before the law was enforced. Additionally, the length of stay in the ER was longer after enforcement. In contrast, the percentage of transfers to other hospitals increased and the length of hospital stay decreased after the LEMD law was enforced. There were no significant differences in consent rates for most LSTs and life-sustaining procedures before and after enforcement; however, the consent rate for ICU admission decreased after enforcement. The rate of survival to discharge was particularly high in the group of patients who had completed advance directives before the ER visit.

The time from ER registration to signing the life-sustaining treatment plan was longer after the enforcement of the LEMD law, and this can be attributed to the peculiarity of the legal system. In the period immediately after enforcement, not many patients had prepared such documents in advance, and many severely ill patients were unconscious at presentation; thus, it took a long time for all family members to come to a unanimous decision. However, after a certain time period, even in the absence of advance directives, the patient's will to withdraw life-sustaining treatments was recorded in the medical record in many cases or more patients informed family members in advance. Thus, it was possible to withdraw life-sustaining treatments at a relatively early stage with a decision statement from only two family members [7]. If the time to finalize consent is prolonged, the length of ER stay also increases, which may cause overcrowding in the ER [13]. This clearly indicates the need for a simplified procedure for preparing a life-sustaining care scheme. As an alternative, there could be a way for patients to appoint a specific guardian (family member or friend) in advance and give them the right to make decisions in critical situations. In Queensland, the Guardianship and Administration Act 2000 allowed competent adults to give an enduring power of attorney regarding health matters to any person of their choice (family member or friend), allowing the selected person to make decisions in case patients lose their decision-making abilities. If necessary, the Guardianship Tribunal can appoint a guardian if no person has been selected by the patients themselves [14]. This concept is not yet considered appropriate in the context of the traditional culture in Korea, but it may become more suitable in future.

In this study, the increase in the length of ER stay can be understood in relation to the increase in the rate of direct transfer from the ER to other hospitals. Before the LEMD law was implemented, the rate of transfer to a secondary hospital was only 8.2% and that of transfer to a nursing hospital was 1.0%, but the corresponding rates increased to 21.2% and 9.7%, respectively, after implementation (Table 3). Considering that the length of ER stay increased similarly in the group of patients who prepared advance directives in advance, longer stay in the ER is a separate issue from the time required to complete the consent form. The increase in the number of direct transfers to other hospitals, especially nursing hospitals, is the most likely cause for this result. In South Korea, transfer to nursing hospitals is mostly possible during regular consultation sessions, and patients have to wait until regular hours; therefore, selection of a nursing hospital takes up an inordinate amount of time. An increase in the length of ER stay causes overcrowding [13]. This can lead to a reduction in the ER's capacity to accommodate patients, disruption of medical practice for those in urgent need of treatment, and an increase in the mortality rate [15]. A previous study suggested the use of alternative care facilities as one of the ways to resolve ER overcrowding. This has been shown to not only reduce waiting times and medical expenses but also improve patient outcomes and satisfaction with the medical staff [15]. In other words, rather than staying in the ER while waiting for hospitalization or transfer to other hospitals, direct admission to the hospital’s hospice ward could be a good option for patients who have already prepared advance directives or a life-sustaining treatment plan. However, in our hospital, we have no choice but to keep patients waiting in the ER due to the absence of a hospice ward; thus, this area has scope for further improvement.

We predicted that if the number of patients who prepared advance directives or a life-sustaining treatment plan increases, the number of patients discharged to nursing hospitals for palliative care or who can receive home care will increase, and the length of hospital stay will be shortened [16]. This was consistent with the results from our study.

As the disease progresses, the hospitalization rate of patients increases exponentially, which leads to spending more time in the hospital during the last 6 months of life; this is especially true for cancer patients [17]. In cancer patients, nutrition support or pain control is often the most common reason for hospital admission, in addition to chemotherapy administration [18]. However, these types of symptom control treatments can be adequately provided in secondary and nursing hospitals. From a medical professional’s point of view, hospital beds must be used for patients who absolutely need treatment in tertiary university hospitals, but this is not the case in real-world practice as patients and their families demand hospitalization at a tertiary university hospital even for receiving pain control treatment or supportive care. Of course, such requests are rational considering the psychological reassurance related to admission to a hospital equipped with doctors and facilities competent enough to cope with emergencies; however, admission to a tertiary university hospital solely for receiving supportive care can deprive other cancer patients who require more invasive and urgent treatment.

Especially, in the case of hospitals without a hospice ward, this problem is more serious. A study showed that receiving specialized treatment in the ambulatory care sector is associated with reduced bed utilization and a reduced need for hospitalization, especially in advanced cancer patients [17]. Therefore, if patients who have already prepared advance directives or a life-sustaining treatment plan receive care in a nursing hospital or primary medical institution, the frequency of unplanned hospitalizations in a higher-level hospital can be reduced, and other patients can avail treatment opportunities. Furthermore, from the patient’s point of view, receiving treatment at a specialized facility with caregivers who can provide focused hospice care can reduce emotional and mental stress [19]. As mentioned above, this could be of great help in improving the patient's quality of life [20]. Therefore, it can be inferred that a reduction in the length of hospital stay is an important consequence of the enactment of the LEMD law and fulfills the purpose of the enforcement of this law.

When patients fill out orders for LST, advance directives, or the life-sustaining treatment plan, they may refuse all ultimate treatments; these forms can generally be seen as a means of refusing all life sustaining treatments, but some life-sustaining treatments can be selected and received as per the patient's will. Based on previous research, it was expected that the consent rates for each treatment would differ before and after the implementation of the LEMD law [21]. However, this study showed that there was no difference in the consent rates to perform CPR, intubation, and CRRT, although the consent rate for ICU admission showed differences before and after enforcement.

Previous international studies have revealed that even if a life-sustaining treatment plan has been prepared, treatment goals vary widely, subjectively reflecting each patient’s will. For example, there are patients who receive palliative care only, patients who receive some restrictive treatment, and patients who receive all treatments other than CPR [22, 23]. However, the procedures described in the life-sustaining treatment plan (CPR, intubation, CRRT, and ICU admission) are interconnected. Furthermore, in critical situations, it is not easy for patients’ guardians to choose only one or two life-sustaining treatment options. Besides, there are differences in the scope of treatments that patients want to receive depending on the timing of preparation of the life-sustaining treatment plan [24]. For instance, if a patient visits the ER and prepares a life-sustaining treatment plan, there is insufficient time to think about how the treatment can be continued. Moreover, considering the situation in which a life-sustaining treatment plan is being prepared, it is conceivable that a plan prepared in the ER is intended for a patient who is not likely to be resuscitated or is dying. In such situations, it is extremely difficult to explain the treatments related to the patient’s resuscitation to the patient’s guardians, who are in despair, and select some of them. Additionally, even if guardians select only some of the listed procedures, it may not make sense for medical professionals to perform only some of those treatments for the aforementioned reasons. Therefore, there seems to be no difference in the rate of the procedures described in the life-sustaining treatment plan before and after the implementation of the LEMD law.

The rate of survival to discharge was significantly higher in patients who had prepared advance directives before visiting the ER than in patients who prepared the life-sustaining treatment plan in the ER. This suggests that patients who had prepared advance directives before visiting the ER had time to thoroughly ponder about the quality of their remaining life. Earlier studies have also revealed that the preparation of a letter of intent for life-sustaining treatments in advance is associated with decreased hospital mortality and an increase in hospice use [25], which is equivalent to an increase in the rate of survival to discharge. Thus, the patients in this study who prepared advance directives before visiting the ER wanted an improved quality of life at home or in a hospice facility rather than life-sustaining treatments at a university hospital. Although the number of patients in this group was small and not statistically significant, the reduction in the length of hospital stay by more than half can be considered a supporting result. Since the enactment of the LEMD law, an increasing number of patients are writing their advance directives beforehand; thus, in the future, it is necessary to improve the quality of medical services for patients receiving palliative care by implementing domestic hospice programs.

Previously, there was no legal system for the suspension of life-sustaining treatments, and thus, life-sustaining treatments were continued regardless of the patient’s will. Alternatively, in cases in which the patient was on the verge of death, the designated guardian filled out the orders for LST. However, with the implementation of the LEMD law, patients have been given the legal right to refuse life-sustaining treatments. Near the end of life, intensive care for patients who do not want life-sustaining treatments imposes physical, emotional, and financial burdens [26, 27], which lead to a poor quality of life. Therefore, to improve the quality of life of patients with a short life expectancy, it is necessary to respect the freedom and will of patients who have prepared a life-sustaining treatment plan. This approach will also reduce unnecessary admissions to tertiary university hospitals, enabling the provision of high-quality treatment to other patients who need intensive treatment.

Since the implementation of the LEMD law in South Korea, there have been no studies comparing the effects of this law and orders for LST (a similar document used before implementation), although the number of patients who prepare advance directives or a life-sustaining treatment plan is increasing. Therefore, this study has a significant advantage in that it is the first to investigate the differences in the consent for orders for LST before and after the enforcement of the LEMD law.

There are several limitations to this study. First, this study was conducted at one tertiary university hospital in South Korea, and thus, the results cannot be generalized to the entire country. In subsequent research, continuing the study in several other university hospitals will increase the reliability of the results and contribute to generalization. Second, we only included those who had prepared orders for LST or advance directives before visiting the ER (outpatient/other institutions) or prepared orders for LST or a life-sustaining treatment plan in the ER; thus, patients who completed the document in the ward after admission through the ER were not included. In the follow-up study, it would be interesting to include patients who completed the consent form in the ward after hospitalization. Third, since the formats for orders for LST and advance directives or a life-sustaining treatment plan are different, some detailed procedures were excluded from the study (Table 1).

## Conclusion

The length of stay in the ER after the enforcement of the LEMD law was lengthened both for patients who had prepared advance directives before visiting the ER and those who completed a life-sustaining treatment plan in the ER. In contrast, after the implementation of the LEMD law, the length of stay in the tertiary university hospital decreased for patients who prepared their LST plans in the ER. Moreover, the consent rate for ICU admission decreased. However, the consent rates to perform CPR, intubation, and CRRT were not significantly different before and after the enforcement of the LEMD law.

## Data Availability

The datasets used and/or analyzed during the current study are available from the corresponding author on reasonable request.

## Abbreviations

CPR: Cardiopulmonary resuscitation
CRRT: Continuous renal replacement therapy
DNR: Do-not-resuscitate
ER: Emergency room
ICU: Intensive care unit
LEMD: Life Extension Medical Decision
LST: Life-Sustaining-Treatment
